# Association of Lung Cancer and Tea-Drinking Habits of Different Subgroup Populations: Meta-Analysis of Case-Control Studies and Cohort Studies

**Published:** 2019-09

**Authors:** Zijun GUO, Mei JIANG, Wenting LUO, Peiyan ZHENG, Huimin HUANG, Baoqing SUN

**Affiliations:** Guangzhou Institute of Respiratory Disease, The First Affiliated Hospital of Guangzhou Medical University, Guangzhou, China

**Keywords:** Tea, Meta-analysis, Case-control studies, Cohort studies, Lung cancer

## Abstract

**Background:**

We aimed to investigate the association between lung cancer and tea-drinking habits of different subgroup populations.

**Methods:**

Systematic search of the PubMed, Web of Science, China National Knowledge Infrastructure (CNKI) and Sinomed databases from database construction until January 2017 for English and Chinese language articles on association of lung cancer and tea drinking. Meta-analysis was used to calculate the combined odds ratio (OR) value and its 95% confidence interval (95% CI). The Newcastle-Ottawa scale was used to evaluate the quality of the studies and Q-test and I_2_ was used for heterogeneity testing.

**Results:**

Forty two papers were included, 30 case-control studies included 14578 lung cancer patients and 180574 controls, 12 cohort studies included 543825 subjects, of which the outcome was 5085 with lung cancer. Tea drinkers were found to have a decreased OR of lung cancer compared with non-tea drinkers (OR 0. 80, 95% CI: 0. 73, 0. 87). Consumption of green, black or unspecified tea has a protective effect compared with not drinking tea at all. Increased intake of green tea to 7. 5 g per day can further reduce the OR of lung cancer (OR 0. 69, 95% CI: 0. 48–0. 98). Tea consumption had a protective effect against lung cancer in non-smokers, Further analysis found that drinking of one or more cups of tea a day has a protective effect on smokers (OR 0. 79, 95% CI: 0. 64–0. 96).

**Conclusion:**

Tea drinking could be a protective factor in lung cancer.

## Introduction

Currently, lung cancer is one of the malignant cancers in the world with the highest incidence and mortality rates (1). Therefore, the prevention of lung cancer is of utmost importance. Many studies have investigated the risk of lung cancer and tea consumption, but the conclusions were not consistent (2–4). A meta-analysis in 2009 (5) found that drinking green tea has a protective effect on lung cancer statistically, while there was no association between drinking black tea and lung cancer. Either black or green tea consumption have a protective effect on lung cancer statistically. Hence there is a controversy between the results of these two studies (6).

Smoking is a major risk factor for lung cancer (7, 8). In vivo animal experiments have shown that tea polyphenols can decrease the probability of tumor formation and decrease the size and peak proliferation of tumors (9, 10). When smoking cessation is difficult, whether tea drinking can antagonize the effects of smoking on lung cancer risk is important in the prevention of lung cancer. Intake of green tea can decrease the lung cancer risk in smoking populations (11). However, two previous systematic meta-analyses did not find that tea drinking can decrease the risk of contracting lung cancer in smoking populations.

This study collected all local and overseas published articles up till January 2017 to carry out a meta-analysis to investigate the association between tea intake in different subgroup populations and lung cancer.

## Methods

Tea, green tea, black tea, lung cancer, lung neoplasm, lung tumor, and lung carcinoma were used as keywords to search in the PubMed, Web of Science, the China National Knowledge Infrastructure (CNKI) and Sinomed databases. The keywords were used together or individually to search all databases from database construction until January 2017. The literature search was performed independently by two authors. All articles must fulfill the following inclusion criteria: 1) Lung cancer; 2) Case-control studies or cohort studies; 3) Exposure risk factors involves tea drinking, and study contains either OR or relative risk (RR), and its 95% CI, or these values can be computed.

Data extraction and quality assessment: The first author, publication year, study period, region, type of study, type of controls, sample size (number of cases and controls), tea drinking status, adjusted OR or RR and its 95% CI, were extracted from every article. The Newcastle-Ottawa scale (NOS) was used to evaluate the quality. Data extraction and quality assessment were also performed independently by two authors.

Statistical analysis: RevMan 5. 3 software was used for statistical analysis and the OR values and 95% CI comparing either tea drinking or highest tea intake with non-tea drinking were obtained from combining various studies. The amount of tea intake was shown by the weight of tea leaves (in grams). The intake amount in this study was readjusted and one cup of tea was defined as 2. 5 g of tea leaves (2).

The Q-test and I_2_ was used for heterogeneity testing, both P<0. 1 and I_2_>50% defined as the presence of heterogeneity (12). When heterogeneity presented, subgroup analysis was carried out to eliminate heterogeneity; and if heterogeneity still exists, sensitivity analysis was carried out and each study was omitted individually to see if there were studies with significant effects on heterogeneity. If heterogeneity was still presented, the random effects model was used for statistical analysis. A funnel plot was constructed to investigate publication bias (13), and an asymmetrical funnel plot shows that there is publication bias.

## Results

### Basic information

**Fig. 1:**
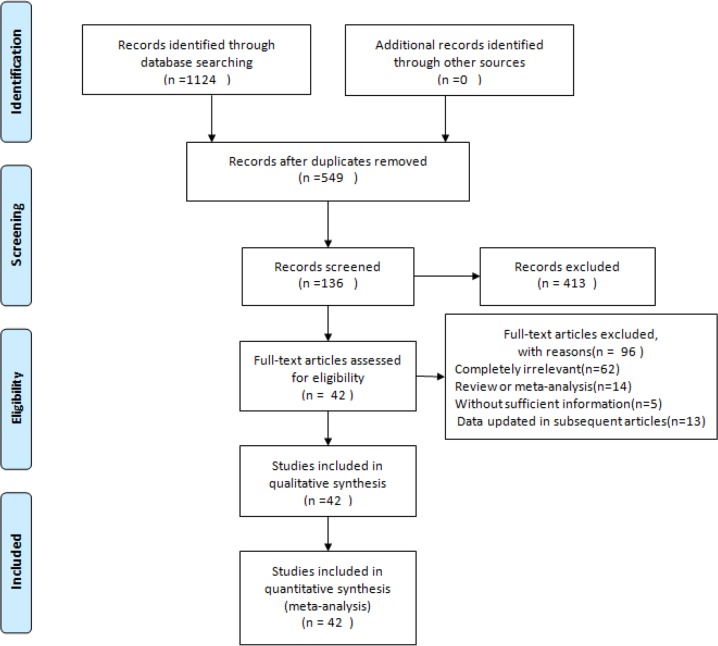
Process followed in the selection of studies

The initial search yielded 549 articles. Through screening of titles and abstracts, 413 articles were excluded and 60 articles were selected for data extraction after careful reading of the article. As the data from 13 articles were repeated in subsequent studies, these studies were excluded. Complete data could not be extracted from five studies and these studies were also excluded. Finally, 42 studies were included in the meta-analysis in this study (3–4, 14–53) (Fig. 1). There were 19, 433 lung cancer patients and 718, 854 controls. 30 case-control studies, with 17 population-based case-control studies, one mortality-based case-control study and the remainder were hospital-based case-control studies. Case-control studies included 14578 lung cancer patients and 180574 controls. Twelve cohort studies included 543825 subjects, of which the outcome was 5085 with lung cancer. Two studies investigated the association between lung cancer and black and green tea consumption, 12 studies for green tea and seven for black tea. The remaining 21 studies did not specify the type of tea (Table 1).

**Table 1:** Characteristics of published studies on tea consumption and lung cancer risk

***Study***	***Study period***	***Country***	***Study design***	***Case-control or cohort***	***Tea type***	***OR (95%CI)***	***NOS score (stars)***
Romain 2016(14)	1996–2001	Canada	PCC	1111/1469	Black	0. 71[0. 61, 0. 83]	8
Wu 2015(15)	2001–2010	China	PCC	117/1196	Green	0. 87[0. 70, 1. 07]	7
Wang 2015(16)	2012–2014	China	HCC	88/84	Tea	0. 97[0. 53, 1. 76]	7
Mai 2015(17)	1992–2011	US	Cohort	1137/94887	Tea	0. 83[0. 71, 0. 96]	8
Katarzyna 2015(18)	2014	Poland	PCC	92/156	Green	0. 49[0. 26, 0. 93]	7
Bao 2014(18)	2010–2013	China	HCC	50/50	Green	0. 22[0. 08, 0. 60]	7
Rup 2014(20)	2009–2012	India	PCC	230/460	Tea	0. 95[0. 61, 1. 49]	7
P. gnagna 2013(22)	2004–2005	Italy	Cohort	178/4158	Tea	0. 72[0. 52, 0. 99]	8
Xu 2013(21)	2006–2012	China	HCC	1225/1234	Tea	0. 98[0. 84, 1. 15]	7
Yumie 2013(23)	2002–2009	China	Cohort	359/60733	Tea	0. 66[0. 53, 0. 83]	7
Jin 2013(24)	2003–2010	China	PCC	1424/4543	Green	1. 05[0. 92, 1. 20]	8
Yumiel 2013(25)	2001–2011	China	Cohort	428/70839	Tea	1. 00[0. 81, 1. 23]	7
Lin 2012(26)	2004–2008	China	HCC	170/340	Green	0. 34[0. 21, 0. 55]	7
Zhang 2012(27)	1997–2009	China	PCC	900/133811	Tea	1. 16[1. 01, 1. 32]	8
Bganesh 2011(28)	1997–1999	India	HCC	408/1383	Tea	0. 24[0. 11, 0. 55]	6
Jiang 2011(29)	2009–2011	China	HCC	100/100	Tea	0. 92[0. 53, 1. 61]	7
Lu 2009(30)	1992–1995	US	Cohort	201/38207	Tea	0. 81[0. 61, 1. 08]	7
Han 2008(31)	2003–2008	China	HCC	523/1924	Green	0. 56[0. 44, 0. 73]	7
Zhang 2008(32)	2002–2006	China	PCC	505/529	Tea	1. 16[0. 89, 1. 53]	8
Wang 2008(24)	2006	China	HCC	363/363	Tea	0. 60[0. 44, 0. 82]	7
Qli 2008(33)	1994–2001	Japan	cohort	302/41138	Green	1. 29[0. 98, 1. 69]	8
Yan 2008(35)	1999–2004	US	PCC	558/837	Green&Black	0. 52[0. 42, 0. 66]	7
Tao 2007(36)	2002–2006	China	HCC	47/94	Tea	0. 72[0. 31, 1. 70]	6
Shinchi 2006(4)	1995–2005	Japan	Cohort	222/16247	Green	1. 13[0. 82, 1. 56]	8
Hu 2002(39)	1994–1997	Canada	PCC	161/483	Tea	0. 52[0. 34, 0. 81]	8
Mattew 2005(37)	1995–1996	China	PCC	122/121	Green	0. 83[0. 44, 1. 54]	7
Ja 2005(38)	1982–1998	US	PCC	993/986	Black	0. 95[0. 79, 1. 13]	6
Nagano 2001(41)	1979–1994	Japan	Cohort	395/35930	Green	0. 86[0. 66, 1. 12]	9
Zhong 2001(40)	1992–1994	China	PCC	649/675	Green	0. 97[0. 74, 1. 26]	7
Hivonen 2001(42)	1995–1998	Finland	PCC	791/25643	Tea	0. 66[0. 53, 0. 81]	7
Kei 2000(3)	1986–1997	Japan	Cohort	69/9483	Green	1. 01[0. 62, 1. 63]	6
Ki 1997(45)	1992–1993	China	HCC	105/105	Tea	0. 50[0. 23, 1. 10]	7
Fredrik 1998(43)	1989–1995	Sweden	PCC	124/235	Black	1. 23[0. 78, 1. 96]	8
Maria 1998(44)	1994–1996	Uruguay	HCC	427/428	Black	0. 78[0. 60, 1. 02]	7
Alexandra 1996(46)	1986–1990	Netherlands	Cohort	764/120088	Black	0. 58[0. 49, 0. 70]	8
Zheng 1996(47)	1986–1993	US	Cohort	312/35057	Black	0. 78[0. 62, 0. 99]	7
Gosta 1996(48)	1989–1993	Sweden	PCC	308/504	Black	0. 71[0. 53, 0. 94]	7
Xu 1996(49)	1987–1993	China	PCC	598/926	Tea	0. 84[0. 68, 1. 03]	9
Ohno 1995(50)	1988–1991	Japan	PCC	333/666	Tea	0. 57[0. 39, 0. 83]	9
Tewes 1990(51)	1981–1983	China	PCC	200/200	Green&Black	0. 98[0. 66, 1. 45]	6
Mettlin 1989(52)	1982–1987	US	HCC	569/569	Tea	0. 71[0. 56, 0. 91]	6
Kinlen 1988(53)	1969–1986	UK	Cohort	718/12868	Tea	1. 67[1. 31, 2. 13]	7

PCC, population-based case-control study; HCC, hospital-based case-control study; US, United States; UK, United Kingdom

The quality evaluation scores of every article ranged from 6 to 9 points. Among these articles, 36 were high-quality articles (NOS 7–9) and the remaining articles were medium-quality articles (NOS 6) (Table 1).

### Association of tea drinking and lung cancer

**Fig. 2:**
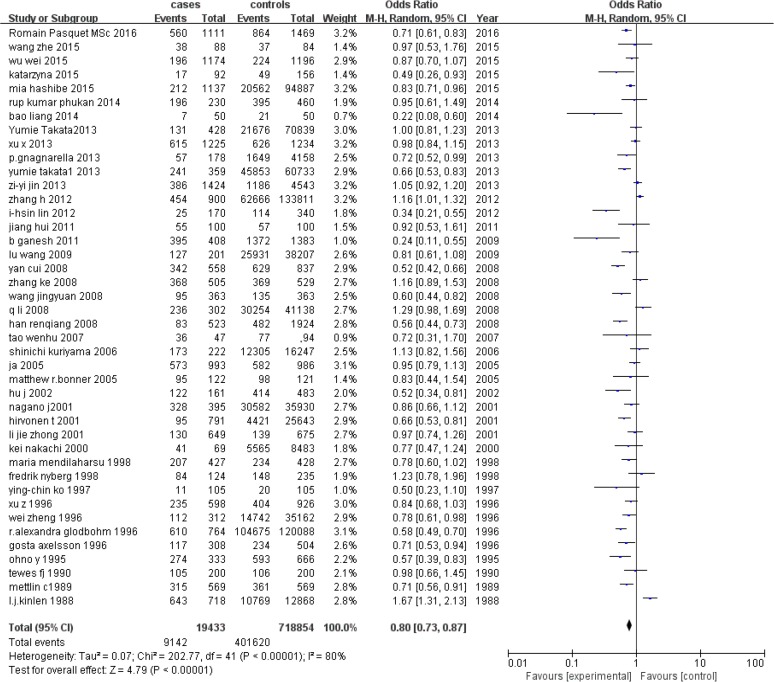
Association between tea consumption and OR for lung cancer

**Fig. 3:**
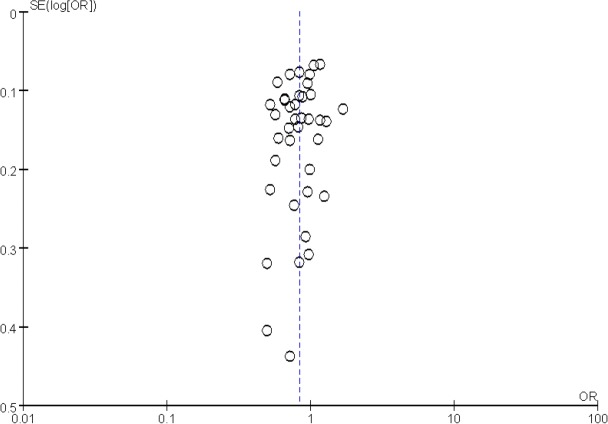
Funnel plot of studies on tea consumption and lung cancer

When compared with non-tea drinking populations, tea drinking was found to have a protective effect against lung cancer (OR 0. 80, 95% CI: 0. 73–0. 87) (Fig. 2). Statistically significant heterogeneity was observed (I_2_=80%, *P*<0. 01) (Fig. 3). Subgroup analyses were done in order to identify sources of heterogeneity. As shown in Table 2, the heterogeneity was not reduced by subgroup analysis of Tea types, Study design, Geographical region, Sex, Smoking status and Study period. When stratified analysis was conducted by study design. It was found to have a decreased OR in the case-control studies (OR 0. 76, 95% CI: 0. 68, 0. 85), but no statistically significant association in cohort studies (OR 0. 88, 95% CI: 0. 74, 1. 05).

**Table 2:** Subgroup analyses of tea intake and lung cancer risk

***Study***	***Number (n)***	***OR (95%CI)***	***Case-control or cohort(n)***	***Heterogeneity test***	
				***I2(%)***	***P-value(%)***
All studies	42	0. 80[0. 73, 0. 87]	19433/718854	80	<0. 01
2. 5g/day	25	0. 79[0. 68, 0. 91]	10932/404166	82	<0. 01
Cohort	10	0. 89[0. 71, 1. 11]	4888/394604	87	<0. 01
CC	15	0. 71[0. 58, 0. 87]	6044/9562	79	<0. 01
7. 5g/day	16	0. 82[0. 67, 1. 01]	7652/277373	86	<0. 01
Cohort	6	0. 87[0. 60, 1. 28]	2470/234754	93	<0. 01
CC	10	0. 91[0. 70, 1. 18]	4904/54490	80	<0. 01
Tea types					
Green tea	14	0. 75[0. 61, 0. 92]	5750/111640	84	<0. 01
Cohort	4	1. 02[0. 81, 1. 28]	988/101798	51	0. 1
CC	10	0. 79[0. 73, 0. 86]	4762/9842	86	<0. 01
2. 5g/day	9	0. 73[0. 54, 0. 98]	1959/104387	76	<0. 01
Cohort	4	1. 00[0. 87, 1. 15]	1511/103722	49	0. 1
CC	5	0. 41[0. 21, 0. 80]	971/2589	73	<0. 01
7. 5g/day	7	0. 69[0. 48, 0. 98]	1667/103926	84	<0. 01
Cohort	4	0. 86[0. 74, 0. 98]	988/101798	90	<0. 01
CC	3	0. 61[0. 44, 0. 85]	679/2128	59	0. 09
Black tea	9	0. 80[0. 70, 0. 91]	4797/159909	65	<0. 01
Cohort	2	0. 78[0. 72, 0. 84]	1076/155250	72	0. 05
CC	7	0. 82[0. 76, 0. 90]	3721/4659	45	0. 09
2. 5g/day	7	0. 88[0. 68, 1. 14]	4039/158872	87	<0. 01
Cohort	2	0. 76[0. 44, 1. 29]	1076/155250	90	<0. 01
CC	5	0. 94[0. 72, 1. 23]	2963/3622	79	<0. 01
7. 5g/day	5	0. 75[0. 56, 1. 02]	2805/157168	78	<0. 01
Cohort	2	0. 81[0. 49, 1. 36]	1077/155250	67	0. 08
CC	3	0. 68[0. 40, 1. 16]	1728/1918	73	0. 02
Tea unknown	21	0. 84[0. 73, 0. 96]	8627/316770	78	<0. 01
Cohort	4	0. 77[0. 70, 0. 86]	2056/254560	0	0. 39
CC	17	0. 89[0. 83, 0. 95]	7526/119181	76	<0. 01
2. 5g/day	9	0. 75[0. 59, 0. 96]	4934/140907	84	<0. 01
Cohort	4	0. 86[0. 56, 1. 32]	2824/137556	92	<0. 01
CC	6	0. 67[0. 58, 0. 78]	2110/3351	20	0. 29
7. 5g/day	5	1. 13[0. 81, 1. 57]	4258/27690	83	<0. 01
Cohort	1	1. 67[1. 31, 2. 13]	718/12868	-	-
CC	4	1. 01[0. 71, 1. 43]	2462/3411	79	<0. 01
Study design					
Cohort	12	0. 88[0. 74, 1. 05]	5085/538740	84	<0. 01
CC	30	0. 76[0. 68, 0. 85]	14578/180574	78	<0. 01
Geographical region					
Western population	15	0. 81[0. 70, 0. 94]	7325/329216	79	<0. 01
Cohort	6	0. 93[0. 74, 1. 16]	3310/299387	83	<0. 01
CC	9	0. 73[0. 61, 0. 88]	4015/29829	73	<0. 01
Asian population	25	0. 80[0. 70, 0. 92]	10630/321441	80	<0. 01
Cohort	5	0. 94[0. 91, 0. 98]	1416/172637	91	<0. 01
CC	20	0. 96[0. 94, 0. 99]	9214/148804	75	<0. 01
Sex					
Male	11	0. 82[0. 64, 1. 05]	5183/240914	90	<0. 01
Cohort	4	1. 00[0. 61, 1. 61]	1980/150566	94	<0. 01
CC	7	0. 73[0. 55, 0. 98]	3203/90348	87	<0. 01
Female	14	0. 80[0. 67, 0. 95]	4447/304808	64	<0. 01
Cohort	5	0. 93[0. 82, 1. 06]	1105/228461	19	0. 29
CC	8	0. 90[0. 82, 0. 97]	3073/76121	28	0. 21
Smoking status					
Smoking	8	0. 80[0. 63, 1. 01]	3663/32347	79	<0. 01
Cohort	2	0. 67[0. 56, 0. 81]	969/29801	0	0. 65
CC	5	0. 85[0. 63, 1. 15]	2694/2546	80	<0. 01
No-smoking	8	0. 67[0. 51, 0. 89]	2973/74512	81	<0. 01
Cohort	1	0. 66[0. 53, 0. 83]	359/60733	-	-
CC	7	0. 63[0. 46, 0. 85]	2545/3673	77	<0. 01
Study period					
Before 2000	22	0. 80[0. 70, 0. 91]	9660/326269	77	<0. 01
Cohort	7	0. 91[0. 67, 1. 23]	2761/291876	89	<0. 01
CC	15	0. 76[0. 67, 0. 85]	6899/34393	57	<0. 01
After 2000	15	0. 75[0. 64, 0. 89]	7422/147484	81	<0. 01
Cohorts	2	0. 87[0. 63, 1. 20]	606/74997	66	<0. 01
CC	13	0. 74[0. 61, 0. 91]	6457/11754	83	<0. 01

CC, case-control study

### All subgroup analysis by study design

#### Type of tea

Green, black or unspecified tea were correlated with protection against lung cancer in the case-control studies. Black tea and tea unknow also showed protective effect in cohort studies (Table 2).

There were no statistical significances in consumption of more than one cup/day black tea and lung cancer. Increasing daily intake of green tea to 7. 5 g increased the protective effect against lung cancer both in case-control studies and Cohort studies (Table 2).

#### Geographical region

There were obvious differences in the protective effect of tea drinking on lung cancer of Western and Asian countries in different study designs (Table 2).

#### Gender

Both females and males, tea drinking had a protective effect against lung cancer the case-control studies (Table 2). But no statistically significant association was found in cohort studies.

#### Study period

In both time periods of studies conducted before 2000 and after 2000, tea drinking showed a protective effect against lung cancer in the case-control studies. But no statistically significant association in cohort studies (Table 2).

#### Smoking status

**Fig. 4:**
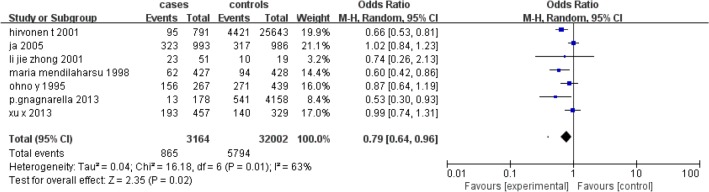
Association of between 2. 5g/day tea consumption and risk for lung cancer on smoking status

Tea consumption has a protective effect against lung cancer in non-smoking populations. When daily tea intake was greater than 2. 5 g, there was a protective effect of tea drinking on lung cancer in smoking populations (Fig. 4). All studies showed heterogeneity but no publication bias (I2=63%, *P*=0. 01).

## Discussion

This study showed that tea drinking had some protective effect against lung cancer. Increasing amounts of green tea intake showing a further decrease in lung cancer OR. Black tea also showed a protective effect of against lung cancer, but it didn’t further decrease the OR of lung cancer by increasing the amount. This can be attributed to the differences in the production of the two tea (49). The main active component in green tea, EGCG was present in higher amounts in green tea than black tea. This could explain why increasing black tea consumption didn’t increase its protective effect against lung cancer.

In smoking populations, when increased tea consumption to 2. 5 g/day, it showed a protective effect against lung cancer, which was consistent with previous studies (9). The preventive effect on lung cancer by tea could be due to the presence of polyphenols in tea. Evidence has shown that EGCG can prevent the formation of mutated cells and that EGCG can increase the activity of phase II enzymesin vivo animal studies (54–57). Phase II enzymes are involved in the detoxification of carcinogens that will be subsequently excreted (58). EGCG could induce apoptosis in cells that were damaged by carcinogens in cigarette smoke (59–61). However, smoking is considered as chronic exposure and long-term smoking has a much greater effect on lung cancer risk than just cumulative effects of daily smoking (62). Hence, long-term intake of high EGCG doses is required to reduce the damage caused by tobacco carcinogens. The types of tea involved in this study are complex, and there was no adjustment for amount of smoking, period of smoking, period of tea drinking, etc. Hence, It need for well-designed studies with larger sample sizes and better control of various confounding factors, and the inclusion of intervention and mechanistic studies, in order to more accurately verify the association of lung cancer and different amounts of different tea in smoking populations.

It showed heterogeneity in this study. Subgroup analysis of sex, smoking status, type of tea, intake amounts and other adjustment factors could not reduce the heterogeneity. The study by Kinlen et al. (53) is the source of heterogeneity when study type, region, sex and study period were used as subgroups. This study had a NOS score of 7, with large number of cases and low sensitivity, and removing it from inclusion did not cause any obvious differences in results. Therefore, the random effects model was used for data analysis in this study.

In addition, The combination of results of studies with different designs (case-control and cohort) lead to biased results, the subgroup analysis by study design of tea types (green tea, black tea and tea un-know), geographical region, sex, smoking status, study period and the amount of tea also have shown different. However, cohort study reveals a causal relationship, and case-control cannot, cohort studies are considered preferable to case-control studies in the hierarchy of scientific evidence, and Cohort studies results should play as the standard. Our results showed that significant association existed in case-control studies, but not in cohort studies. The results may be related to the difference of study design types and sample size. Participants in case-control studies were greatly less than participants included in cohort studies.

The results of this meta-analysis were limited by some factors. Firstly, some articles did not specify the type of tea. Secondly, the data from included studies were raw primary data and most studies were retrospective case-control studies that could have possible bias and confounding factors. Lastly, this study included a small number of countries such as China, Japan and the USA, etc., and the representation by these countries requires further verification. Despite these limitations, our study collected all studies published to date on the association of tea drinking and lung cancer for a meta-analysis, and results showed that tea drinking could have protective effect against lung cancer. Increasing the amount of green tea intake to 7. 5 g a day showed an increased protective effect of green tea against lung cancer. Regular intake of one cup of tea or more could antagonize the effects of smoking on lung cancer in smokers. However, larger sample sizes or prospective cohort studies are required for verification of these results and for further mechanistic studies.

## Ethical considerations

Ethical issues (Including plagiarism, informed consent, misconduct, data fabrication and/or falsification, double publication and/or submission, redundancy, etc.) have been completely observed by the authors.
